# Optimization of total protein and activity assays for the detection of MMP-12 in induced human sputum

**DOI:** 10.1186/1471-2466-10-40

**Published:** 2010-08-02

**Authors:** Peter LaPan, Jeff Brady, Christal Grierson, Margaret Fleming, Doug Miller, Joe Sypek, Bin Fu

**Affiliations:** 1 Pfizer Global Research and Development, Department of Biological Technologies, 35 Cambridge Park Drive, Cambridge, MA, 02140, USA; 2 Translational Medicine Research Collaboration Laboratory, James Arrot Drive, Ninewells Hospital, Dundee, DD1 9SY, UK; 3 Pfizer Global Research and Development, Department of Inflammation, 200 CambridgePark Drive, Cambridge, MA, 02140, USA; 4 Pfizer Global Research and Development, Department of Discovery Translational Medicine, 500 Arcola Road, Collegeville PA, 19426, USA

## Abstract

**Background:**

Proteolysis of matrix components, in particular elastin, is a major contributing factor to the development of lung diseases such as emphysema and chronic obstructive pulmonary disease (COPD). MMP-12 (macrophage elastase) is a protease known to be involved in the progression of lung disease. The relatively low abundance of MMP-12 has precluded the development of quantitative assays that can accurately measure MMP-12 protein levels and activity across cohorts of healthy and diseased individuals.

**Methods:**

Commercial antibodies were screened for performance in sandwich ELISA and capture FRET activity assay formats. Precision, accuracy, sensitivity, dilution linearity, and spike recovery were evaluated using sputum samples.

**Results:**

Total protein and capture FRET activity assays were developed that were sensitive enough to detect MMP-12 in 37 of 38 donor sputum samples. A comparison of results between the two assays shows that a majority of sputum MMP-12 is in the active form. No differences were seen between normal, asthmatic, and COPD donors.

**Conclusion:**

Sensitive and quantitative assays for both MMP-12 activity and total protein in human induced sputum have been developed. These assays can be used to evaluate MMP-12 as a biomarker for lung disease, and to monitor efficacy of potential therapeutic compounds.

## Background

MMP-12, also know as macrophage elastase, is a member of the matrix metalloproteinases. MMP-12 is synthesized as a 55 kD precursor molecule which is then processed into a 22 kD mature active form by serine proteinases [1]. MMP-12 activity is regulated through a balance of inhibitory interactions with the Tissue Inhibitors of Metalloproteinases (TIMPs) and activating interactions with proteases including other MMPs [2]. Active MMP-12 has the capacity to degrade extracellular matrix components, in particular elastin [3], and is involved in matrix remodeling both in normal development and during pathophysiological disease. Disruption of the balance between MMP-12 and its endogenous inhibitors can result in diseases such as emphysema and COPD.

A role for MMP-12 in cigarette smoke induced emphesyma has been demonstrated [4]. The mechanism involves the induction of TNF-alpha release from macrophages followed by endothelial cell activation, neutrophil recruitment and secretion of matrix degrading proteases [5]. The resulting elastin fragments have been shown to chemotactic for monocytes, resulting in further recruitment and amplification of the inflammatory cascade [6].

A protective role for MMP-12 inhibitors has been postulated [7], but specific, sensitive assays for the detection of MMP-12 in human induced sputum are currently unavailable. A challenge to the development of MMP-12 assays is the autocatalysis seen when MMP-12 is purified away from its natural inhibitors, the TIMPs. To ascertain the value of MMP-12 as a biomarker, as well as to monitor the efficacy of small molecule therapeutics targeting MMP-12 directly, we have developed both an activity and total MMP-12 protein assay that are capable of monitoring MMP-12 protein levels and activity in human induced sputum.

## Methods

### Antibodies

A total MMP-12 ELISA was developed using the Dendritics (Lyon, France) antibody cat# DDX0284 as a capture antibody and cat # DDX0281 as a detection antibody [8]. The capture antibody for the FRET assay, cat# DDX0282, was also obtained from Dendritics. Additional MMP-12 specific antibodies were tested from Sigma (St. Louis, MO) cat# M0807, R&D Systems (Minneapolis, MN) cat# MAP 919 and MAB 917, Genway, (San Diego, CA) cat# 15-288-066A, Genetex (Irvine, CA) cat# GTX11613, Epitomics (Burlingame, CA) cat# 1906-1, Dendritics cat# DDX0280, Affinity Bioreagents (Rockford, IL) cat# PA1-25216 and PY-10169, Abgent (San Diego, CA) cat#AB6196a, Abcam (Cambridge, MA) cat# ab38395, ab38910, ab38908, ab37718, ab11614, ab11613, and ab0025.

### Antibody conjugation

Conjugation of AP (alkaline phosphatase) to the detection antibody DDX0281 was accomplished via a stable thioether linkage between maleimide coupled to AP and a sulfhydryl group introduced onto the antibody. 60 μL of AP (~ 1 mg, Biozyme AP cat# 1596AA2x, 18.59 mg/ml, 111000 U/ml) was dialyzed against 500 mls of buffer EDB (30 mM TEA, 3 M NaCl, 50 μM ZnCl_2_, 1 mM MgCl2 pH 7.6) at 2-8°C overnight. 600-700 ul (~ 0.5 mg) of the monoclonal antibody DDX0281 was dialyzed against 500 mls of TSE pH 8.4 (100 mM TEA, 0.9 mM EDTA, 100 mM NaCl). The protein concentration was measured post dialysis.

AP was activated by incubation with a 38:1 molar ratio of sSMCC (Pierce cat# 1B110605) for 40 minutes at room temperature on rollers. Conversion of primary amines on the antibody was accomplished by incubation with a 300:1 molar ratio of 2-IminoThiolane (Pierce cat# 1L114603). Both reactions were terminated by the addition of excess glycine.

Desalting for both proteins was performed on by gel-filtration on a 5 ml Hi Trap Desalt (G25) column on an AKTA FPLC. The antibody was exchanged into the TSE pH 7.3 buffer by the gel-filtration and the AP was run with TSMZ pH 7.2 (100 mM TEA, 100 mM NaCl, 1 mM MgCl_2_, 50 μM ZnCl_2_). Absorbance at 280 nm was monitored and fractions corresponding to the excluded protein were collected and pooled.

Fractions containing the antibody (~300 μg) and AP (~600 μg) were pooled for an equivalent 2:1 molar ratio of enzyme to antibody. The pool was incubated for 90 mins at room temperature and the reaction was stopped with 40 μL of a 10 mg/ml maleimide solution.

The conjugate was concentrated in a Perbio 7 K concentrator following the manufacturer's instructions, including a water wash to remove glycerol from the membrane. The final volume loaded on a HiTrap column was 700 μl and the conjugate was desalted into TSMZ pH 7.2 at 5 ml/min collecting 500 μl fractions. From the chromatogram monitored at 280 nm fractions 5,6, and 7 were pooled. The yield was 1.5 ml of conjugate solution, with an absorbance of 0.36 AU at 280 nm (~500 ug total). A volume of 50 μl was removed and frozen for analysis before addition of an equal volume of Surmodics Alk Phos Stabilzyme reagent. The final solution containing 160 ug/ml conjugate was stored at 2-8°C.

### FRET substrates

FRET 520 substrate III (cat# 60570-01, Anaspec, San Jose CA, 0.1 mg peptide) was dissolved in 80 μL DMSO. 275 μL of the peptide/DMSO solution was brought to 11 mL with FRET assay Buffer (50 mM Hepes, 100 mM NaCl, 5 mM CaCl_2_, 0.005% Brij-35, pH 7.4) for a final concentration of 20 μM. Additional substrates tested were FRET 520 substrate II (cat# 60569-01), IV (cat# 60571-01), V (cat# 60572-01), VII (cat# 60574-01), IX (cat# 60576-01), XI (cat# 60578-01), XII (cat# 60579-01), XIII (cat# 60580-01), XIV (cat# 60581-01), XV (cat# 60582-01), and XVI (cat# 60583-01) all purchased from Anaspec, (San Jose CA).

### MMP Protein

Recombinant Pro-MMP-12 residues 1 to 470 was supplied by R&D Systems, (Minneapolis, MN) 20 μL total volume at a concentration of 500 μg/mL. A volume of 180 μl TCNB buffer (50 mM Tris pH 7.5, 10 mM CaCl_2_, 0.15 M NaCl, 0.05% Brij35) was added to create a 50 μg/mL stock, which was then aliquoted into 2.5 μL aliquots and stored at -80°C.

To prepare active MMP-12, full length Pro-MMP-12 was diluted in TCNB buffer to 50 μg/mL and allowed to incubate for 24 hours at 37°C. The resultant autocatalyzed MMP-12 was aliquoted into 2.5 μL aliquots and stored at -80°C.

Additional MMPs tested were MMP-1 (cat# 901-MP-010), MMP-2 (cat# 902-MP-010), MMP-3 (cat# 513-MP-010), MMP-7 (cat# 907-MP-010), MMP-8 (cat# 908-MP-010), MMP-10 (cat# 910-MP-010), MMP-13 (cat# 511-MM-010), MMP-14 (cat# 918-MP-010) all supplied by R&D Systems. MMP-9 was manufactured by Pfizer Research (Cambridge, MA). MMP propeptides were diluted to 50 ug/mL in TCNB buffer and treated with 1 mM APMA at 37°C for 2 hours for activation. MMP-14 was diluted to 40 ug/mL and incubated with 0.86 μg/mL furin for 90 minutes at 37°C.

### Batch Preparation of Standard Curves

For both the activity and total protein assays, to increase inter-assay precision of the standard curve, standard dilutions were batch prepared in advance and frozen in aliquots.

To batch prepare prealiquoted standard curves for the activity assay, an aliquot of autocatalyzed MMP-12 was thawed and 2 μL added to 5 mL BlottoB (Rockland Immunochemicals, Philadelphia, PA, cat# B553-0500) in the first channel of a 12 channel pipette reservoir, producing the first standard at 20,000 pg/mL. Seven addition channels are filled with 3 mL BlottoB. 1.5 mL of the 20,000 pg/mL standard is three fold serially diluted with 3 mL of BlottoB to produce six additional standards of 6,667, 2,222, 741, 247, 82, and 27 pg/mL, leaving the eighth channel as the zero standard. A multichannel pipettor is used to transfer 240 μL of each standard into an eight tube PCR strip tube (VWR cat# 82006-606) with attached caps. Aliquots are stored frozen at -80°C.

To batch prepare prealiquoted standard curves for the MMP-12 total protein assay, an aliquot of full length MMP-12 was thawed and 2 μL added to 400 μL PBS 0.05% Tween-20 (137 mM NaCl, 2.7 mM KCl, 10 mM Sodium Phosphate dibasic, 2 mM Potassium Phosphate monobasic, pH of 7.4) to create a 250 ng/mL stock. 38.4 μL of the 250 ng/mL stock was added to 6 mL of ESD100 (CellTechnologies, Mountain View, CA, cat# ESD100-2) supplemented with 50 μg/mL IIR (Immunoglobulin Inhibiting Reagent, Bioreclamation Inc., Westbury, NY. cat# 6LD1074) in the first channel of a 12 channel pipette reservoir, producing the first standard at 1,600 pg/mL. Seven additional channels are filled with 3 mL ESD100+IIR. 3 mL of the 1,600 pg/mL standard is two fold serially diluted with 3 mL of ESD100+IIR to produce six additional standards of 800, 400, 200, 100, 50, and 25 pg/mL, leaving the eighth channel as the zero standard. A multichannel pipettor is used to transfer 240 μL of each standard into an eight tube PCR strip tube with attached caps. Aliquots are stored frozen at -80°C.

### Sample aquisition

Human sputum was collected by Analytical Biological Services (Wilmington, DE) using a protocol originally described by Pavord[9]. An ultrasonic nebuliser was filled with 3% saline and subjects inhaled the nebulised solution for 7 minutes following pre-treatment with inhaled salbutamol. Next the nebuliser was filled with 4% saline and the nebulised solution is inhaled for 7 minutes. Finally, the nebuliser was filled with 5% saline and the nebulised solution was inhaled for 7 minutes. At 5-minute intervals the inhalation was stopped to allow expectoration into a polypropylene container. A total of four expectorations were collected and pooled. The sample was kept on ice until centrifugation to harvest the soluble phase of sputum. The collection was not performed on subjects with an FEV1 (Forced Expiratory Volume in 1 second) <1 L.

Post collection, an equal volume of Buffer A (0.1%DTT, 10% Sputolysin; 1 part Sputolysin and 9 parts normal saline) was added to the sputum sample. The sample was vortexed gently 3 times, and incubated in a 37°C water bath shaker for 15 minutes at 160 RPM. Samples were mixed every 5 minutes during incubation to ensure complete homogenization. Following incubation, samples were centrifuged at 1200 RPM for 10 minutes. Supernatant was removed and aliquoted into 150 μL aliquots before storage at -80°C. Reported concentrations of MMP-12 were not adjusted for this two fold dilution with Buffer A.

### Automated plate washing

Plate washing was performed with a Biotek (Winooski, VT) ELx405 96 channel plate washer. The standard wash protocol involves five 300 μL washes, no soaking, with a crosswise aspiration after the final wash. The soak protocol involves five 300 μL washes, with a 30 second soak for each wash followed by a crosswise aspiration.

### MMP-12 Total Protein ELISA protocol

1. Coat the capture antibody, Dendritics cat# DDX0284, 1 hour at 3 μg/mL, 100 μL/well, 37°C in ELISA coating buffer (0.1 M carbonate/bicarbonate pH 9.55-9.65).

2. Wash with PBS wash buffer (137 mM NaCl, 2.7 mM KCl, 10 mM Sodium Phosphate dibasic, 2 mM Potassium Phosphate monobasic, 0.05% Tween-20, pH 7.4) at RT, standard wash program.

3. Block 1 hour with 200 μL/well Superblock (Pierce, Rockford, IL cat# 37515), 37°C.

4. Wash with PBS wash buffer at RT, standard wash program.

5. Add samples and standards in a total volume of 100 μL/well. Sputum samples are diluted 1:8 in ESD100 (Cell Technology, Inc. Mountain View, CA) supplemented with IIR (Immunoglobulin Inhibitory Reagent cat# 6LD1074, Bioreclamation Inc., Hicksville, NY); incubate overnight at 4°C.

6. Wash with Tris wash buffer (25 mM Tris pH 7.5, 150 mM NaCl, 0.05% Tween 20) at RT using the soak program.

7. Add AP-DDX0281 conjugate detection antibody at 100 ng/mL prepared in Tris wash buffer for 1 hour at RT.

8. Wash with Tris wash buffer at RT using the soak program.

9. Use Invitrogen cat#19589-019 ELISA amplification system. Reconstitute the substrate and amplifier with 12 mL of appropriate diluent 10 minutes at RT in advance of use.

10. Add 50 μL per well of reconstituted substrate, incubate 30 minutes at RT.

11. Add 50 μL per well of reconstituted amplifier in the same sequence as the substrate. Incubate 30 minutes at RT.

12. Add 50 μL 2N sulfuric acid per well.

13. Read OD 495 nm at RT.

### MMP-12-FRET activity assay

1. Dilute the capture antibody to 2 μg/mL in ELISA coating buffer.

2. Add 100 μL per well to a NUNC Starwell plate (Nunc, Roskilde, Denmark cat# 441653).

3. Seal and incubate at RT for 2 hours with agitation.

4. Wash the plate on the ELx405 plate washer, standard wash program with PBS-0.05% Tween-20.

5. Add 250 μL per well of Superblock blocking reagent.

6. Seal and incubate at RT for 2 hours with agitation.

7. Wash the plate on the ELx405 using the standard wash program with PBS-0.05% Tween-20.

8. Add 100 μL per well of standard or unknown diluted in BlottoB.

9. Seal and incubate overnight at 4°C with agitation.

10. Reconstitute the FRET substrate. -Per tube of substrate add 80 μL DMSO. -Dilute 275 μL of stock substrate into 11 mL FRET Assay buffer

11. Wash the plate on the ELx405, standard wash program with PBS-0.05% Tween-20.

12. Blot on paper towels. Apply a black plate sealer (Perkin Elmer, Waltham, MA, cat# 6005189) to the bottom of the Starwell plates.

13. Add 100 μL of substrate solution to each well. Seal with a plate sealer.

14. Incubate the plate at RT for 24 hours with agitation.

15. Remove the plate sealer and place into a fluorescent plate reader.

Read at room temperature, single endpoint, 1 second integration per well, 0.5 second delay between wells, excitation wavelength 485 nm, emission wavelength 527 nm, normal beam.

### Data Analysis

Limits of quantitation are defined as the lowest and highest standards that have both an intra- and inter-assay %CV and %Bias of less than 20%. Dilution linearity is evaluated by calculating the %bias of a sample that is serially diluted. Two or more consecutive serial two fold dilutions exhibiting a %bias less than 20% identify a range of linearity.

To evaluate intra-assay imprecision, three aliquots of each sputum sample are run in duplicate per analytical run. Mean and standard deviations are calculated for each aliquot (N = 3). A total of three analytical runs are performed to assess inter-assay imprecision. Mean and standard deviation are calculated using plate averages for each sputum sample (N = 3).

Curve fitting is performed using the five-parameter logistic fit provided in SoftMax Pro v5.2 (Molecular Devices, Sunnyvale, CA).

## Results

### MMP-12 Total ELISA development

An antibody pair originally identified by Demedts et al. [8] was used in a sandwich format to detect total MMP-12 in human induced sputum. In the original format, antibody DDX0284 is used as a capture antibody and biotinylated antibody DDX0281 is the detection antibody, followed by streptavidin-HRP. This format exhibited poor dilution linearity and poor sensitivity, with only 31 of 38 donor sputum samples above the lower limit of quantitation (LLOQ) of 78 pg/mL when diluted 1:2 (data not shown). A screen of commercially available diluents identified ESD-100 as a diluent that exhibited good dilution linearity in the 1:4 to 1:8 dilution range. Tetrazolium amplification combined with direct alkaline phosphatase conjugation provided enhanced sensitivity, which allowed for a 1:8 dilution of sputum samples. Given an LLOQ of 50 pg/mL and an ULOQ of 1600 pg/mL, the reportable range of the assay is 400-12,800 pg/mL. Under these conditions 37 of 38 donor samples were within the reportable range of the assay.

### Calibration Curve Range

The mean intra-assay imprecision (%CV) and accuracy (%Bias) from five (5) analytical runs for each of the seven calibrators are shown in Additional file 1, Table S1. The inter-assay imprecision (%CV) and accuracy (%Bias) of the calibration curve is shown in Additional file 1, Table S2. The LLOQ of the assay is 50 pg/mL at which concentration the inter-assay %CV and %Bias were 7.7% and -5.8%, respectively. The ULOQ of the assay was 1600 pg/mL at which concentration the inter-assay %CV and %Bias were 2.6% and -1.1%, respectively. A typical calibration curve is shown in Additional file 2, Figure S1.

### Determination of Sample Minimum Required Dilution and the Reportable Range

To determine the optimal dilution factor for human induced sputum, samples were analyzed following a 1:2, 1:4, 1:8, or a 1:16 dilution in ESD100 diluent supplemented with 50 ug/mL IIR. Additional file 1, Table S3 shows that samples run at 1:2 through 1:16 reported similar dilution corrected values, however at 1:16, one of four samples was below LLOQ and at 1:2 one sample was above the ULOQ. To minimize the possibility of diluting samples below the LLOQ, samples are diluted 1:8 before analysis. Based on the LLOQ of 50 pg/mL and the ULOQ of 1,600 pg/mL, the reportable range of MMP-12 in human induced sputum is 400 to 12,800 pg/mL.

### Donor Survey in the Presence and Absence of IIR

To determine the range of MMP-12 concentrations in human induced sputum, and to determine the effect of IIR, 38 induced sputum samples were collected from asthma, COPD and normal donors (Additional file 1, Table S4). Aliquots were analyzed in ESD100 diluent supplemented with 50 μg/mL IIR or ESD100 without IIR.

Results in Additional file 1, Table S5 show that the percent difference in calculated MMP-12 concentrations between samples with and without IIR ranged from -139% to 92%, with 27 of 38 samples showing an absolute difference greater than 25%. Intra-assay %CV without IIR is 49.6%, and with IIR 4.1%. Given the beneficial effect upon intra-assay reproducibility, and to reduce the incidence of false positive signals due to human anti-mouse antibodies, we decided to incorporate IIR into the diluent at a final concentration of 50 μg/mL.

Sputum MMP-12 concentrations assayed in the presence of IIR varied from one sample below the LLOQ to 12,294 pg/mL. The mean value of samples above the LLOQ was 2967 pg/mL with and CV of 4.1% (Additional file 1, Table S5). Samples falling below the LLOQ were not used in calculating mean or SD.

### Analytical Performance: Intra- and Inter-assay Imprecision of the QC Validation Samples

The overall performance of the assay was determined by the intra- and inter-assay imprecision of the validation samples analyzed in three analytical runs. Due to variability seen in two donors initially analyzed, a total of 10 donor samples ranging from 555 to 4154 pg/mL were selected for analysis. The mean intra-assay imprecision (%CV) for these validation samples ranged from 1 to 18% (Additional file 1, Table S6). The inter-assay imprecision (%CV) of the validation samples ranged from 10-23% (Additional file 1, Table S7; n = 3 aliquots for each validation sample analyzed in duplicate). Because none of the endogenous QC validation samples were at the high end of the standard curve, three aliquots of a spike-in sample of 8,000 pg/mL were analyzed in three different runs (Additional file 1, Table S8). The average interpolated concentration for the QC high validation sample was 6,487 pg/mL. Intra- and inter-assay precision of the QC high validation sample are 4.8% and 7.3% respectively.

### Determination of Assay Accuracy and Recovery

MMP-12 protein from R&D Systems was spiked into eighteen human sputum samples and into assay diluent at three different concentrations (final theoretical spiking concentrations of 800, 3200, and 8000 pg/mL). The measured MMP-12 concentration in assay diluent yielded the baseline comparison for recovery in sputum (Additional file 1, Table S9). The actual spiked (nominal) concentration of MMP-12 in sputum samples for the theoretical spiking concentrations of 800, 3200, and 8000 pg/mL was 910, 3814, and 9217 pg/mL respectively. The recovery ranged from 64% to 144% for the high spike sample, 71% to 136% for the mid spike sample, and 81% to 130% for the low spike sample. 38 of 53 determinations were in the range of 75-125% recovery.

### Sample Stability

Percent change from baseline (1 F/T) after freeze thaw ranged from 31% to -10.7% (Additional file 1, Table S10). All donors showed small changes from freeze thaw to freeze thaw with the exception of donor 152, which showed a large change between 1 (baseline) and 2 F/T, and small changes (<5%) thereafter. These results suggest that total MMP-12 is relatively stable to freeze thaw cycles.

The stability of total MMP-12 in induced sputum incubated at RT and 4°C for 2, 4, or 24 hours was evaluated using four donor samples (Additional file 1, Table S11). MMP-12 was stable in sputum with only 2 samples showing >25% change, both samples being 24 hour room temperature samples.

It is suggested that samples should be thawed and held at 4C for no more than 2 hours. Up to 4 freeze thaw cycles have minimal impact on recovery of total MMP-12.

### MMP-12 FRET activity assay

A FRET activity assay makes use of a capture antibody specific for the enzyme of interest coated onto a solid substrate. When a biological sample is applied to the coated antibody, enzyme is captured and other matrix components are washed away. The amount of enzyme captured is proportional to the concentration of the enzyme in the biological fluid. The captured enzyme then cleaves a supplied peptide which tethers a quencher to a fluor, preventing fluorescence. When the peptide is cleaved by the catalytic activity of the enzyme, the fluor will be released from the quencher and fluoresce in proportion to the captured enzyme.

**Figure 1:**
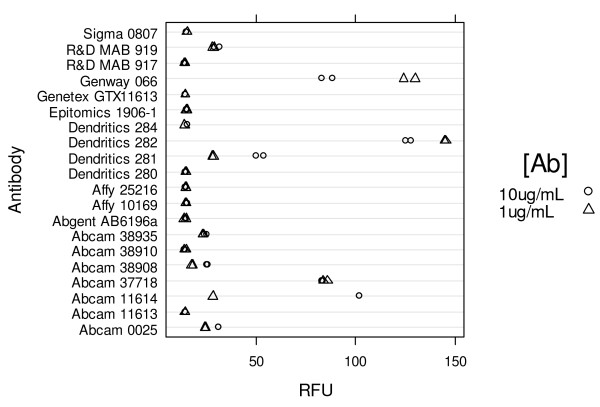
**A comparison of commercially available MMP-12 antibodies as capture antibodies in the FRET activity assay format**.

MMP-12 is the least abundant of human MMP's in sputum, being present at 1/1000 fold the concentration of the more abundant MMPs such as MMP-8 and MMP-9 (data not shown). The specificity of an assay that depended on the intersection of the specificity of the antibody and the FRET peptide would not work for MMP-12, given that any cross reactivity of the antibody with other MMPs, in particular MMP-8 and MMP-9, would saturate the capture antibody binding sites on the plate and reduce sensitivity for MMP-12. We decided that the specificity of the assay must be conferred by the antibody, allowing for the use of a promiscuous FRET peptide. Given this strategy, we screened a panel of antibodies looking at the 24 hour endpoint signal (Figure 1) using 20 μM Substrate V and 10 ng/mL mature MMP-12. Antibodies were coated at either 1 or 10 μg/mL. The highest signal was seen with antibodies from Dendritics (cat# DDX0282) Genway (cat# 15-288-066A) and Abcam (cat# 11614). These antibodies were further tested for specificity against a panel of mature MMPs.

**Figure 2:**
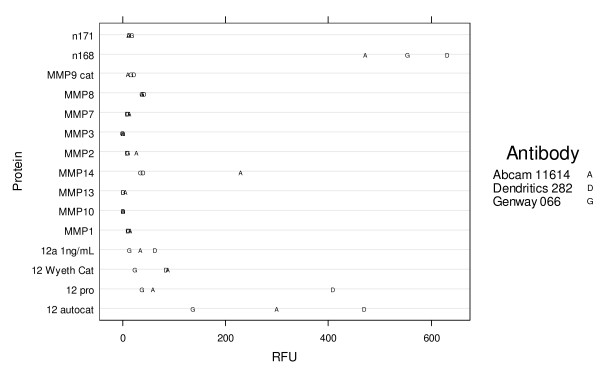
**A plot of RFU (abscissa) vs. analyte (ordinate) in the capture FRET format using antibodies Dendritics 282 (cat# DDX0282), Abcam 11614, or Genway 066 (cat# 15-288-066A)**. Analytes are present at 1 ug/mL, except for 12a - autocatalyzed R&D Systems MMP-12 at 1 ng/mL, 12 Wyeth cat - recombinant catalytic MMP-12 at 10 ng/mL, 12 pro - pro form MMP-12, R&D Systems and 12 autocat - autocatalyzed R&D Systems MMP-12 both at 10 ng/mL. n171 and n168 are two sputum samples with unknown MMP-12 concentrations.

The antibodies of interest were coated at their optimal concentration, 10 μg/mL for Abcam 11614, and 1 μg/mL for Dendritics 282 and Genway 066 as determined from figure 1. Anaspec substrate III was used at a concentration of 20 uM. Analytes (Figure 2) were present at 1 μg/mL, except for 12a (autocatalyzed R&D Systems MMP-12 at 1 ng/mL), 12 Wyeth cat (recombinant catalytic MMP-12 at 10 ng/mL), 12 pro (Pro form MMP-12, R&D Systems) and 12 autocat (autocatalyzed R&D Systems MMP-12) both at 10 ng/mL and n171, n168, two sputum samples with unknown MMP-12 concentrations. The Dendritics antibody DDX0282 was shown to produce the highest signal with mature MMP-12 while showing no cross reactivity with other MMPs (Figure 2).

**Figure 3:**
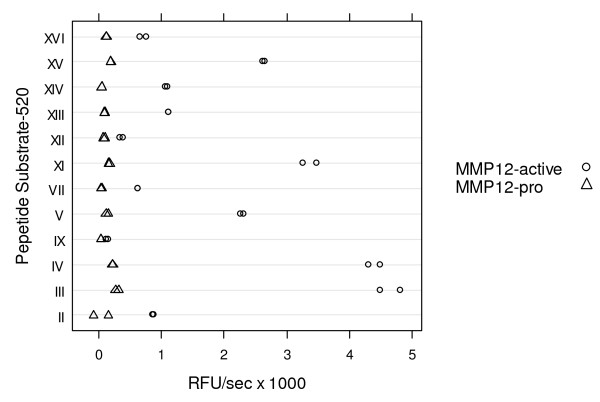
**Vmax (abscissa) is plotted against Anaspec substrate (ordinate) in a solution phase assay using 20 μM substrate and 100 ng/mL pro or autocatalyzed MMP-12**.

In parallel, catalytic MMP-12 was tested against a panel of FRET substrates. Substrate III provided the greatest Vmax and was used in all subsequent assays (Figure 3).

### Calibration Curve Range

The mean intra-assay imprecision (%CV) and accuracy (%Bias) from five (5) analytical runs for each of the seven calibrators are shown in Additional file 1, Table S12. The inter-assay imprecision (%CV) and accuracy (%Bias) of the calibration curve is shown in Additional file 1, Table S13. The LLOQ of the assay was 82 pg/mL at which concentration the inter-assay %CV and %Bias were 9.4% and -16.7%, respectively. The ULOQ of the assay was 20,000 pg/mL at which concentration the inter-assay %CV and %Bias were 0.3% and -0.2%, respectively. A typical calibration curve is shown in Additional file 3, Figure S2.

### Determination of Sample Minimum Required Dilution and the Reportable Range

To determine the optimal dilution factor for human induced sputum, samples were analyzed following a 1:2, 1:4, 1:8, or a 1:16 dilution in BlottoB. Additional file 1, Table S14 shows that samples run at 1:2 through 1:16 reported similar values, however at 1:16, two of four samples were below LLOQ. To minimize the possibility of diluting samples below the LLOQ, samples are diluted 1:2 before analysis. Based on the LLOQ of 82 pg/mL and the ULOQ of 20,000 pg/mL, the reportable range of MMP-12 in human induced sputum is 164 to 40,000 pg/mL.

### Donor Survey

To determine the range of MMP-12 concentrations in human induced sputum, 38 induced sputum samples were collected from asthma, COPD, and normal donors (Additional file 1, Table S4). Sputum MMP-12 concentrations varied from below the LLOQ (1 donor) to 9,535 pg/mL (Additional file 1, Table S15). The mean value of samples above the LLOQ was 1778 pg/mL for normal donors, 2498 pg/mL for asthmatics, and 1298 for COPD donors. The CVs were 9.0, 7.2, and 5.9 respectively. Samples falling below the LLOQ were not used in calculating mean or SD.

### Analytical Performance: Intra- and Inter-assay Imprecision of the QC Validation Samples

The overall performance of the assay was determined by the intra- and inter-assay imprecision of the validation samples analyzed in three (3) analytical runs. The mean intra-assay imprecision (%CV) for Low, Mid, and High validation samples was 19%, 14% and 4%, respectively (Additional file 1, Table S16). The inter-assay imprecision (%CV) of the validation samples was 11%, 8%, and 21% for Low, Mid, and High, respectively (n = 3 aliquots for each validation sample analyzed in duplicate).

### Determination of Assay Accuracy/Recovery

MMP-12 protein from R&D Systems was autocatalyzed and spiked into four human sputum samples and into assay diluent at three different concentrations (final theoretical spiking concentrations of 10,000, 3000, and 1000 pg/mL). The measured MMP-12 concentration in assay diluent yielded the baseline comparison for recovery in sputum (Additional file 1, Table S17). The actual spiked (nominal) concentration of MMP-12 in sputum samples for the theoretical concentrations of 10,000, 3,000, and 1000 pg/mL was 19,686, 5,828, and 1,786 pg/mL respectively. The recovery ranged from 78.9% to 107.2% for the high spike sample, 83.3% to 99.9% for the mid spike sample, and 86.5% to 107.5% for the low spike sample.

### Sample Stability

The stability of active MMP-12 in induced sputum incubated at RT and 4°C for 2, 4, or 24 h was evaluated using four donor samples (Additional file 1, Table S18). MMP-12 was not stable in sputum with most samples showing > 10% bias at all time points under both room temperature and 4°C incubation. Changes between time points were not always cumulative, indicating that some of the differences seen were due to assay variability. Neither time nor temperature showed an obvious trend. It is suggested that samples should be thawed and held at 4°C for no more than one hour prior to processing.

Percent change from baseline after freeze thaw ranged from 23.7% to -30.7% (Additional file 1, Table S19). Change from freeze thaw to freeze thaw were not cumulative. Donors showed both increase and decrease in MMP-12 concentration upon repeated freeze thaw. Due to the lack of a consistent trend, it is recommended that samples not be subjected to more than a total of 2 freeze thaw cycles.

### Comparison of total protein and activity levels in healthy and diseased donors

**Figure 4:**
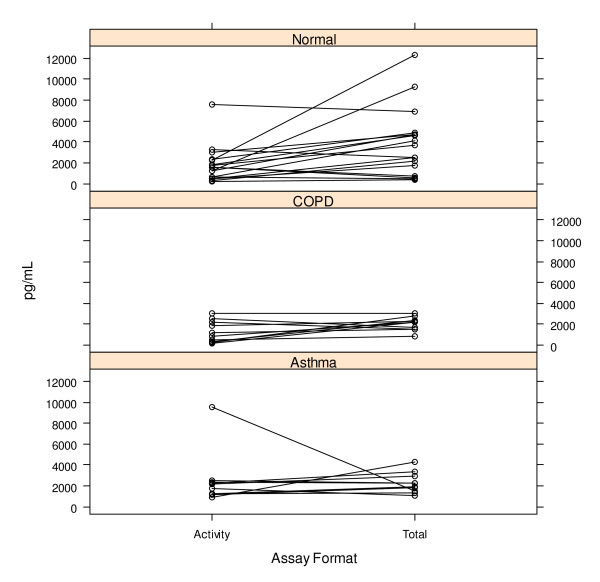
**A pairwise plot of interpolated MMP-12 concentrations for both activity and total MMP-12 protein concentration in normal, COPD, and asthmatic donors**.

**Figure 5:**
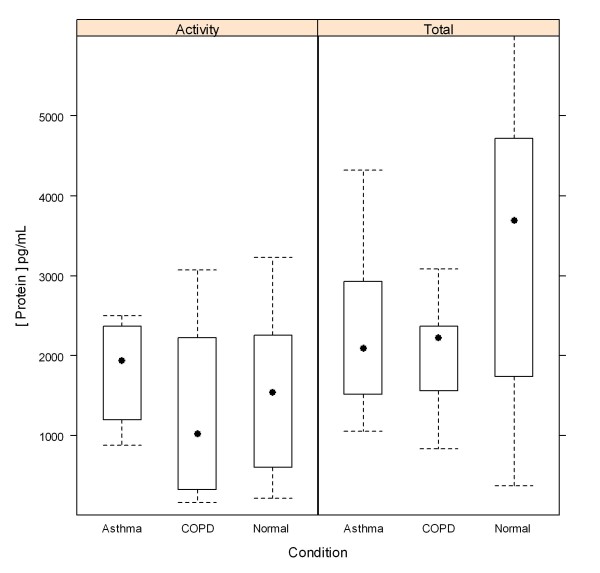
**A boxplot of condition (abscissa) vs interpolated MMP-12 protein concentration (ordinate) trellised by assay format**.

The MMP-12 total protein and activity assays were used to assess total protein and activity levels in induced sputum from 18 healthy, 10 asthma, and 10 COPD donors. Figure 4 shows that interpolated concentrations for MMP-12 are similar for both total protein and activity, suggesting that a majority of the MMP-12 in sputum is in the mature, active form. A comparison of MMP-12 levels across disease states (Figure 5) shows that the differences do not reach significance at the p = 0.05 level (Student's t-test).

## Discussion

Elastin is a major structural component of lung, and elastin degradation is a contributing factor to lung diseases such as emphysema and COPD. Elastin, the primary substrate for MMP-12, is digested at numerous sites generating chemotactic matrikines [3,10]. Both alveolar macrophages [11,12] and bronchial epithelial cells [13,14] serve as pulmonary reservoirs of MMP-12, the latter secreting MMP-12 in response to cigarette smoke.

Evidence citing the involvement of MMP-12 in lung disease is provided both by genetics [15,16] and by functional inhibition of MMP-12 in animal models [17,18]. To investigate the efficacy of novel MMP-12 inhibitors [19], sensitive, accurate assays that can measure MMP-12 total protein and activity in biologically relevant fluids such as sputum are needed.

In this report we describe two fully validated MMP-12 assays, one for the detection of total MMP-12 protein and the second for the detection of MMP-12 activity in human induced sputum. With these assays we have measured total protein and activity in induced sputum from both healthy and diseased (COPD, asthma) donors. We find that the majority of MMP-12 present in sputum is in active form, and we don't see statistically significant differences between healthy and diseased individuals as previously reported by Demedts [8]. However our donor sample is small: 18 normal, 10 COPD, and 10 asthma. In the Demedts COPD cohort, 7 of 28 (25%) of the individuals have MMP-12 protein levels greater than twice the mean for that group. Our results show a mean for the COPD cohort (1297.9 pg/mL) below the mean for the normals (1778.2 pg/mL) with no outliers amongst the COPD patients. Other factors such as normalization methods and differences in sample collection may also contribute to the differences we are seeing with previously published data.

We have screened a panel of ten different MMPs against the peptide substrates shown in figure 3 and found that all peptides are cleaved by at least three different MMPs, and eight of the peptides were cleaved by more than five MMPs (data not shown). Specifically we have found that MMP-9, which is 1000 fold more abundant in sputum than MMP-12 (personal communication, A. Winkler, Pfizer Inflammation) will cleave all of the peptides tested. Given the substrate promiscuity and abundance of MMP-9 in human induced sputum, we felt it was necessary to develop a capture FRET assay where specificity is conferred by the capture antibody. We would expect that a solution phase assay, without the specificity of a capture antibody, would measure primarily MMP-9 activity. Analogous to the total protein assay, our capture FRET activity assay does not detect elevated levels of MMP-12 activity in diseased donors, as has been reported elsewhere [8].

Our current measurements of MMP-12 total protein and activity are normalized to volume. We have experimented with alternative normalization methods such as total protein and cells counts, without seeing differences in relative levels of MMP-12 levels or activity from patient to patient. Studies are currently underway to more thoroughly characterize MMP-12 levels in healthy and diseased populations.

## Conclusions

A total protein and an activity assay for the detection of MMP-12 in human induced sputum have been developed. These assays show good specificity, sensitivity, accuracy and precision and are suitable for the analysis of clinical samples. A study involving 38 donor sputum samples shows that the majority of MMP-12 present in sputum is in the mature, active form.

## Abbreviations

RT: room temperature; C: centigrade; AP: alkaline phosphatase; OD: optical density; TEA: triethanolamine; MMP: matrix metalloproteinase; RPM: revolutions per minute; LLOQ: lower limit of quantitation; ULOQ: upper limit of quantitation; SD: standard deviation; CV: coefficient of variation; cat#: catalog number; FEV: forced expiratory volume

## Competing interests

All authors are employees of Pfizer Global Research.

## Authors' contributions

PL performed experiments, contributed to analysis of the data and drafted the manuscript. JB, CG, and MF performed experiments, made substantial contributions to the interpretation of data, and made important criticisms on the manuscripts intellectual content. DM, JS, and BF made substantial contributions to the interpretation of data, and made important criticisms on the manuscripts intellectual content. All authors read and approved the final manuscript.

## Pre-publication history

The pre-publication history for this paper can be accessed here:

http://www.biomedcentral.com/1471-2466/10/40/prepub

## Supplementary Material

Additional file 1 **Supporting tables**. Data table S1 through S19. Click here for file

Additional file 2 **Figure S1**. An example standard curve for the MMP-12 total protein assay. Click here for file

Additional file 3 **Figure S2**. An example standard curve for the MMP-12 FRET activity assay. Click here for file
