# Supplementary material for: Optimization of total protein and activity assays for the detection of MMP-12 in induced human sputum
Source: BMC Pulm Med. 2010 Aug 2;10:40. doi: 10.1186/1471-2466-10-40 (PMC2921351; doi:10.1186/1471-2466-10-40)
Supplement: Additional file 1 — Supporting tables. Data table S1 through S19. [file 1471-2466-10-40-S1.DOC]

**Additional File 1**

| Table S1 MMP-12 Total ELISA  Intra-assay Imprecision of the Calibration Curve | | | | | | | |
| --- | --- | --- | --- | --- | --- | --- | --- |
| **Calibrator concentration (pg/mL)** | **1600** | **800** | **400** | **200** | **100** | **50** | **25** |
| Avg % CV | 6.8 | 8.0 | 12.3 | 8.7 | 14.2 | 20.3 | 36.8 |
| Avg % bias | -5.8 | -3.9 | -5.0 | -4.7 | -10.1 | -15.1 | -18.7 |
| N=6 for run 1 N=2 for runs 2-5 | | | | | | | |

| Table S2 MMP-12 Total ELISA  Inter-assay Imprecision of the Calibration Curve | | | | | | | |
| --- | --- | --- | --- | --- | --- | --- | --- |
| **Calibrator concentration (pg/mL)** | **1600** | **800** | **400** | **200** | **100** | **50** | **25** |
| **Analytical Run** | **Mean Back-calculated Concentrations From Individual Analytical Runs** | | | | | | |
| PL080625 | 1507.7 | 768.7 | 380.0 | 190.5 | 89.9 | 42.4 | 20.3 |
| PL080624 | 1600.0 | 800.7 | 398.9 | 200.3 | 101.0 | 50.6 | 23.6 |
| PL080623 | 1601.5 | 804.9 | 391.6 | 206.5 | 105.1 | 49.8 | 13.9 |
| PL080628a | 1599.5 | 802.2 | 395.1 | 202.9 | 108.1 | 44.2 | 19.5 |
| PL080701b | 1601.3 | 793.7 | 408.6 | 199.4 | 98.0 | 48.5 | 25.3 |
| Mean | 1582.0 | 794.0 | 394.8 | 199.9 | 100.4 | 47.1 | 20.5 |
| SD | 41.5 | 14.8 | 10.4 | 5.9 | 7.0 | 3.6 | 4.4 |
| % CV | 2.6 | 1.9 | 2.6 | 3.0 | 7.0 | 7.7 | 21.4 |
| % bias | -1.1 | -0.7 | -1.3 | 0.0 | 0.4 | -5.8 | -17.8 |

| Table S3 MMP-12 Total ELISA  Dilution Linearity | | | | | |
| --- | --- | --- | --- | --- | --- |
| **Donor ID** | **diln** | **Average (pg/mL)** | **SD** | **%CV** | **% of previous dilution** |
| 151 | 2 | 2449.7 | 28.3 | 1.2 |  |
|  | 4 | 2486.4 | 76.8 | 3.1 | 101.5 |
|  | 8 | 2869.4 | 148.0 | 5.2 | 115.4 |
|  | 16 | 3365.0 | 59.5 | 1.8 | 117.3 |
| 154 | 2 | 1666.8 | 10.0 | 0.6 |  |
|  | 4 | 1744.5 | 24.0 | 1.4 | 104.7 |
|  | 8 | 2011.7 | 77.6 | 3.9 | 115.3 |
|  | 16 | 2226.0 | 44.3 | 2.0 | 110.7 |
| 163 | 2 | 578.2 | 21.9 | 3.8 |  |
|  | 4 | 682.9 | 7.1 | 1.0 | 118.1 |
|  | 8 | 834.3 | 27.9 | 3.3 | 122.2 |
|  | 16 | NA | NA | NA | NA |
| 190 | 2 | NA | NA | NA |  |
|  | 4 | 4745.1 | 11.7 | 0.2 | NA |
|  | 8 | 5450.5 | 113.5 | 2.1 | 114.9 |
|  | 16 | 7236.4 | 23.1 | 0.3 | 132.8 |

| Table S4 Donor Attributes | | | | | | | | | |
| --- | --- | --- | --- | --- | --- | --- | --- | --- | --- |
| **Condition** | **Donor ID** | **Age** | **Gender** | **Race** | **Smoker** | **How long** | **PPD** | **Date of Diagnosis** | **PFT** |
| Asthma | 151 | 66 | Male | Asian | No |  |  | 1997 |  |
| Asthma | 166 | 69 | Female | Black | No |  |  | 1999 | 2.06 |
| Asthma | 156 | 74 | Female | Caucasian | No |  |  | 1997 | 2.49 |
| Asthma | 163 | 65 | Female | Asian | No |  |  | 1986 | 1.37 |
| Asthma | 170 | 29 | Female | Caucasian | Yes | 10 years | 1-2 cigarettes | 1987 | 2.85 |
| Asthma | 169 | 42 | Female | Caucasian | No |  |  |  | 1.23 |
| Asthma | 154 | 37 | Female | Black | No |  |  |  |  |
| Asthma | 178 | 39 | Female | Black | No |  |  | 2004 | 2.37 |
| Asthma | 182 | 42 | Female | Caucasian | No |  |  |  |  |
| Asthma | 184 | 59 | Female | Black | No |  |  | 1967 |  |
|  |  |  |  |  |  |  |  |  |  |
| COPD | 153 | 39 | Male | Black | Yes | 26 years | 1 |  | 2.81 |
| COPD | 152 | 64 |  | Asian | Yes | 40 years | 1 | 1990 |  |
| COPD | 173 | 67 | Male | Caucasian | Yes | 38 years | 1 |  | 1.08 |
| COPD | 165 | 62 | Male | Caucasian | Yes | 45 years | 1 |  | 1.17 |
| COPD | 155 | 76 | Female | Caucasian | No |  |  |  |  |
| COPD | 176 | 48 | Female | Caucasian | Yes | 30 years | 1.5 |  |  |
| COPD | 180 | 73 | Female | Caucasian | No |  |  |  |  |
| COPD | 181 | 63 | Female | Caucasian | Yes | 40 years | 1 |  |  |
| COPD | 183 | 73 | Male | Caucasian | Yes | 43 years | 2 |  |  |
| COPD | 185 | 61 | Female | Caucasian | Yes | 30 years | 3 cigarettes | 2005 |  |
|  |  |  |  |  |  |  |  |  |  |
| Normal | 150 | 27 | Female | Caucasian | No |  |  |  |  |
| Normal | 167 | 60 | Male | Caucasian | No |  |  |  |  |
| Normal | 164 | 40 | Male | Caucasian | No |  |  |  |  |
| Normal | 158 | 49 | Male | Caucasian | No |  |  |  |  |
| Normal | 171 | 37 | Male | Caucasian | No |  |  |  |  |
| Normal | 174 | 49 | Female | Hispanic | No |  |  |  |  |
| Normal | 157 | 79 | Female | Caucasian | No |  |  |  |  |
| Normal | 168 | 78 | Female | Caucasian | No |  |  |  |  |
| Normal | 175 | 35 | Male | Asian | No |  |  |  |  |
| Normal | 177 | 77 | Female | Caucasian | No |  |  |  |  |
| Normal | 186 | 34 | Female | Causasian | No |  |  |  |  |
| Normal | 187 | 37 | Female | Causasian | No |  |  |  |  |
| Normal | 188 | 42 | Female | Causasian | No |  |  |  |  |
| Normal | 189 | 42 | Male | Causasian | No |  |  |  |  |
| Normal | 190 | 63 | Female | Causasian | No |  |  |  |  |
| Normal | 191 | 57 | Female | Black | No |  |  |  |  |
| Normal | 192 | 76 | Female | Causasian | No |  |  |  |  |
| Normal | 193 | 43 | Male | Causasian | No |  |  |  |  |

| Table S5 MMP-12 Total ELISA | | | | | | | | |
| --- | --- | --- | --- | --- | --- | --- | --- | --- |
| Donor Survey | | | | | | | | |
|  |  | **With IIR** | | **No IIR** | |  |  |  |
| **Condition** | **Donor ID** | **Avg (pg/mL)** | **% CV** | **Avg (pg/mL)** | **% CV** | **ABS(% Difference)** | **% Difference** |  |
| **Asthma** | 151.0 | 2269.0 | 1.0 | 1434.4 | 98.3 | 45.1 | 45.1 |  |
|  | 154.0 | 2254.5 | 0.5 | 1884.7 | 10.9 | 17.9 | 17.9 |  |
|  | 156.0 | 1314.9 | 2.9 | 2546.6 | 11.5 | 63.8 | -63.8 |  |
|  | 163.0 | 1057.4 | 5.4 | 3302.0 | 80.2 | 103.0 | -103.0 |  |
|  | 166.0 | 1928.5 | 1.8 | 3943.3 | 102.4 | 68.6 | -68.6 |  |
|  | 169.0 | 2931.6 | 0.2 | 1403.2 | 100.7 | 70.5 | 70.5 |  |
|  | 170.0 | 1853.0 | 1.2 | 2019.3 | 7.7 | 8.6 | -8.6 |  |
|  | 178.0 | 3395.5 | 0.6 | 2721.3 | 16.2 | 22.0 | 22.0 |  |
|  | 182.0 | 4316.7 | 2.1 | 3461.1 | 77.2 | 22.0 | 22.0 |  |
|  | 184.0 | 1514.1 | 0.5 | 4292.6 | 103.4 | 95.7 | -95.7 |  |
| Average |  | 2283.5 | 1.6 | 2700.9 | 60.9 | 51.7 | -16.2 |  |
| **COPD** | 152.0 | 1557.3 | 1.7 | 1284.4 | 89.2 | 19.2 | 19.2 |  |
|  | 153.0 | 1693.9 | 2.1 | 3162.9 | 65.4 | 60.5 | -60.5 |  |
|  | 155.0 | 2221.8 | 0.3 | 4168.7 | 22.5 | 60.9 | -60.9 |  |
|  | 165.0 | 2366.9 | 1.8 | 1410.4 | 95.0 | 50.6 | 50.6 |  |
|  | 173.0 | 836.6 | 1.1 | 1046.0 | 12.5 | 22.2 | -22.2 |  |
|  | 176.0 | 2783.9 | 2.4 | 1317.1 | 88.3 | 71.5 | 71.5 |  |
|  | 180.0 | 2330.2 | 2.2 | 3243.5 | 60.7 | 32.8 | -32.8 |  |
|  | 181.0 | 3084.8 | 1.3 | 4253.2 | 18.5 | 31.8 | -31.8 |  |
|  | 183.0 | 1555.3 | 5.0 | 1430.5 | 95.5 | 8.4 | 8.4 |  |
|  | 185.0 | 2233.1 | 2.6 | 1002.1 | 2.2 | 76.1 | 76.1 |  |
| Average |  | 2066.4 | 2.1 | 2231.9 | 55.0 | 43.4 | 1.8 |  |
| **Normal** | 150.0 | 367.9 | 7.3 | 1926.2 | 31.1 | 135.9 | -135.9 |  |
|  | 157.0 | 3696.4 | 3.4 | 2178.6 | 31.0 | 51.7 | 51.7 |  |
|  | 158.0 | 6894.0 | 5.7 | 2543.1 | 36.1 | 92.2 | 92.2 |  |
|  | 164.0 | 520.2 | 2.7 | 2909.1 | 65.5 | 139.3 | -139.3 |  |
|  | 167.0 | 4659.0 | 1.0 | 1925.5 | 44.7 | 83.0 | 83.0 |  |
|  | 168.0 | 4663.2 | 3.0 | 1974.2 | 28.2 | 81.0 | 81.0 |  |
|  | 171.0 | 477.5 | 2.7 | 2137.3 | 27.1 | 127.0 | -127.0 |  |
|  | 174.0 | 755.1 | 0.5 | 2521.1 | 36.2 | 107.8 | -107.8 |  |
|  | 175.0 | 4093.3 | 2.7 | 2997.5 | 58.1 | 30.9 | 30.9 |  |
|  | 177.0 | 2544.6 | 1.0 | 1973.3 | 46.6 | 25.3 | 25.3 |  |
|  | 186.0 | 12294.7 | 2.8 | 4836.9 | NA | 87.1 | 87.1 |  |
|  | 187.0 | 4716.4 | 1.0 | 4196.2 | 26.4 | 11.7 | 11.7 |  |
|  | 188.0 | 4878.0 | 1.3 | 5801.4 | 83.4 | 17.3 | -17.3 |  |
|  | 189.0 | NA | NA | 2590.7 | 6.9 | na | na |  |
|  | 190.0 | 9304.4 | 3.8 | 4778.8 | NA | 64.3 | 64.3 |  |
|  | 191.0 | 2202.9 | 2.0 | 4205.7 | 25.6 | 62.5 | -62.5 |  |
|  | 192.0 | 2464.6 | 0.6 | 5551.9 | 76.9 | 77.0 | -77.0 |  |
|  | 193.0 | 1739.6 | 73.5 | 2536.6 | 4.5 | 37.3 | -37.3 |  |
|  |  | 3898.3 | 6.8 | 3199.1 | 39.3 | 72.4 | -10.4 |  |
| **Grand Average** | | **2966.8** | **4.1** | **2813.5** | **49.6** | **59.0** | **-8.7** |  |

| Table S6 MMP-12 Total ELISA  Intra-assay Imprecision of the QC Validation Samples | | | | | | | |
| --- | --- | --- | --- | --- | --- | --- | --- |
|  |  | **Experiment** | | | | | |
|  |  | **PL080729a** | | **PL080730a** | | **PL080731a** | |
| **Donor ID** | **Condition** | **Avg (pg/mL)** | **% CV** | **Avg (pg/mL)** | **% CV** | **Avg (pg/mL)** | **% CV** |
| 151 | Asthma | 2692 | 2 | 2336 | 1 | 2196 | 7 |
| 165 | COPD | 1953 | 6 | 1586 | 1 | 1615 | 7 |
| 168 | Normal | 4333 | 3 | 3643 | 6 | 3637 | 3 |
| 169 | Asthma | 2301 | 13 | 1804 | 8 | 1885 | 3 |
| 170 | Asthma | 892 | 14 | 644 | 14 | 630 | 6 |
| 175 | Normal | 1506 | 7 | 1087 | 2 | 1104 | 5 |
| 180 | COPD | 1016 | 3 | 730 | 5 | 662 | 6 |
| 183 | COPD | 671 | 6 | 462 | 15 | 531 | 18 |
| 186 | Normal | 5124 | 4 | 3814 | 6 | 3522 | 1 |
| 191 | Normal | 1473 | 3 | 1029 | 6 | 1429 | 7 |
| **Average** |  |  | **6** |  | **6** |  | **6** |

| Table S7 MMP-12 Total ELISA  Inter-assay Imprecision of the QC Validation Samples | | | | | | |
| --- | --- | --- | --- | --- | --- | --- |
|  |  | **Experiment ID** | | |  |  |
| **Donor ID** | **Condition** | **PL080729a** | **PL080730a** | **PL080731a** | **Average** | **% CV** |
| 151 | Asthma | 2692 | 2336 | 2196 | 2408 | 11 |
| 165 | COPD | 1953 | 1586 | 1615 | 1718 | 12 |
| 168 | Normal | 4333 | 3643 | 3637 | 3871 | 10 |
| 169 | Asthma | 2301 | 1804 | 1885 | 1997 | 13 |
| 170 | Asthma | 892 | 644 | 630 | 722 | 20 |
| 175 | Normal | 1506 | 1087 | 1104 | 1232 | 19 |
| 180 | COPD | 1016 | 730 | 662 | 803 | 23 |
| 183 | COPD | 671 | 462 | 531 | 555 | 19 |
| 186 | Normal | 5124 | 3814 | 3522 | 4154 | 21 |
| 191 | Normal | 1473 | 1029 | 1429 | 1311 | 19 |
|  |  |  |  |  | **Average** | **17** |

| Table S8 MMP-12 Total ELISA  Intra- and Inter-assay Imprecision of the QC-High Validation Sample | | | | | |
| --- | --- | --- | --- | --- | --- |
| **Experiment ID** | **Aliquot** | **pg/mL** | **Average (pg/mL)** | **Intra-assay Imprecision (%CV)** | **Inter-assay Imprecision (%CV)** |
| PL080922 | 1 | 7408.9 | 6715.3 | 9.2 | 7.3 |
|  | 2 | 6521.3 |  |  |  |
|  | 3 | 6215.7 |  |  |  |
| PL080923 | 1 | 6839.3 | 6804.3 | 1.7 |  |
|  | 2 | 6677.1 |  |  |  |
|  | 3 | 6896.4 |  |  |  |
| PL080924 | 1 | 6181.2 | 5941.7 | 3.6 |  |
|  | 2 | 5878.1 |  |  |  |
|  | 3 | 5765.9 |  |  |  |
| **Average** |  |  | **6487.1** | **4.8** |  |

| Table S9 MMP-12 Total ELISA  Spike Recovery | | | | | |
| --- | --- | --- | --- | --- | --- |
| **Donor ID** | **Endogenous** | **Level** | **Average** | **Nominal** | **% Recovery** |
| 150 | 747 | Hi | 7401 | 9217 | 75 |
|  |  | Low | 1469 | 910 | 93 |
|  |  | Med | 3628 | 3814 | 81 |
| 151 | 4172 | Hi | 18732 | 9217 | 144 |
|  |  | Low | 5332 | 910 | 114 |
|  |  | Med | 9877 | 3814 | 131 |
| 153 | 1847 | Hi | 15608 | 9217 | 143 |
|  |  | Low | 2929 | 910 | 114 |
|  |  | Med | 6561 | 3814 | 120 |
| 156 | 1120 | Hi | 14593 | 9217 | 143 |
|  |  | Low | 2494 | 910 | 130 |
|  |  | Med | 6369 | 3814 | 132 |
| 158 | 7043 | Hi | 17475 | 9217 | 112 |
|  |  | Low | 8945 | 910 | 123 |
|  |  | Med | 12725 | 3814 | 125 |
| 166 | 3089 | Hi | 14506 | 9217 | 121 |
|  |  | Low | 4136 | 910 | 112 |
|  |  | Med | 8138 | 3814 | 123 |
| 167 | 3837 | Hi | 12606 | 9217 | 99 |
|  |  | Low | 3998 | 910 | 92 |
|  |  | Med | 7116 | 3814 | 98 |
| 169 | 2634 | Hi | 12255 | 9217 | 106 |
|  |  | Low | 3559 | 910 | 108 |
|  |  | Med | 6964 | 3814 | 113 |
| 174 | 22 | Hi | 8866 | 9217 | 96 |
|  |  | Low | 1164 | 910 | 125 |
|  |  | Med | 4194 | 3814 | 109 |
| 177 | 693 | Hi | 12790 | 9295 | 129 |
|  |  | Low | 1991 | 924 | 129 |
|  |  | Med | 5359 | 3641 | 126 |
| 178 | 1057 | Hi | 6519 | 9295 | 64 |
|  |  | Low | 1668 | 924 | 89 |
|  |  | Med | 3760 | 3641 | 82 |
| 180 | 682 | Hi | 13459 | 9295 | 136 |
|  |  | Low | 1979 | 924 | 129 |
|  |  | Med | 5625 | 3641 | 132 |
| 181 | 848 | Hi | 9123 | 9295 | 91 |
|  |  | Low | 1721 | 924 | 102 |
|  |  | Med | 4241 | 3641 | 96 |
| 182 | 809 | Hi | 8805 | 9295 | 88 |
|  |  | Low | 1343 | 924 | 81 |
|  |  | Med | 3571 | 3641 | 82 |

| Table S9 MMP-12 Total ELISA  Spike Recovery (Cont’d) | | | | | |
| --- | --- | --- | --- | --- | --- |
| **Donor ID** | **Endogenous** | **Level** | **Average** | **Nominal** | **% Recovery** |
| 184 | 1800 | Hi | 15025 | 9295 | 138 |
|  |  | Low | 2997 | 924 | 118 |
|  |  | Med | 7167 | 3641 | 136 |
| 187 | 3286 | Hi | 13381 | 9295 | 109 |
|  |  | Low | 4227 | 924 | 109 |
|  |  | Med | 7474 | 3641 | 113 |
| 188 | 1547 | Hi | 7044 | 9295 | 66 |
|  |  | Low | 2232 | 924 | 96 |
|  |  | Med | 3589 | 3641 | 71 |
| 193 | 1598 | hi | 9945 | 9295 | 93 |
|  |  | low | 2284 | 924 | 97 |
|  |  | med | 4875 | 3641 | 96 |

| Table S10 MMP-12 Total ELISA  Freeze Thaw Stability | | | | | | | | | | | | |
| --- | --- | --- | --- | --- | --- | --- | --- | --- | --- | --- | --- | --- |
| **Subject #** | 152 | | | 163 | | | 174 | | | 186 | | |
| **Baseline Conc*** | 1951.8 | | | 1009.3 | | | 529.0 | | | 5647.2 | | |
| **F/T Sample Assay Date** | 1-Jul-08 | | | 1-Jul-08 | | | 1-Jul-08 | | | 1-Jul-08 | | |
| **Replicate #** | **2 F/T** | **3 F/T** | **4 F/T** | **2 F/T** | **3 F/T** | **4 F/T** | **2 F/T** | **3 F/T** | **4 F/T** | **2 F/T** | **3 F/T** | **4 F/T** |
| 1 | 2401 | 2432 | 2415 | 951 | 1006 | 960 | 497 | 480 | 494 | 5865 | 6346 | 6977 |
| 2 | 2446 | 2478 | 2686 | 978 | 1020 | 1071 | 451 | 510 |  | 6748 | 5908 | 6855 |
| Mean | 2424 | 2455 | 2550 | 965 | 1013 | 1016 | 474 | 495 | 494 | 6306 | 6127 | 6916 |
| SD | 32 | 33 | 191 | 19 | 10 | 78 | 32 | 22 | NA | 624 | 309 | 86 |
| % CV | 1.3% | 1.3% | 7.5% | 2.0% | 1.0% | 7.7% | 6.8% | 4.4% | NA | 9.9% | 5.0% | 1.2% |
| % change from baseline | 24% | 25.8% | 30.7% | -4% | 0.4% | 0.6% | -10% | -6.4% | -6.6% | 12% | 8.5% | 22.5% |
| * The baseline analyte concentration in pg/mL is obtained from 1F/T sample since fresh is not available. | | | | | | | | | | | | |
|

| Table S11 MMP-12 Total ELISA  Short Term Stability | | | | | | | | | |
| --- | --- | --- | --- | --- | --- | --- | --- | --- | --- |
| **Donor ID** | **Baseline (pg/mL)** | **Temp** | **Time (hours)** | **Replicate 1** | **Replicate 2** | **Average (pg/mL)** | **SD** | **CV** | **% Change** |
| 150 | 823.8 | 22C | 2 | 794.1 | 813.0 | 803.6 | 13.3 | 1.7 | -2.5 |
|  |  |  | 4 | 939.3 | 1084.6 | 1011.9 | 102.8 | 10.2 | 22.8 |
|  |  |  | 24 | 1348.2 | 1343.5 | 1345.9 | 3.4 | 0.3 | 63.4 |
|  |  | 4C | 2 | 1142.7 | 720.3 | 931.5 | 298.7 | 32.1 | 13.1 |
|  |  |  | 4 | 768.3 | 765.3 | 766.8 | 2.1 | 0.3 | -6.9 |
|  |  |  | 24 | 970.6 | 968.7 | 969.6 | 1.4 | 0.1 | 17.7 |
| 156 | 1200.6 | 22C | 2 | 1142.7 | 1184.2 | 1163.5 | 29.3 | 2.5 | -3.1 |
|  |  |  | 4 | 1278.4 | 1308.1 | 1293.3 | 21.0 | 1.6 | 7.7 |
|  |  |  | 24 | 1357.8 | 1304.3 | 1331.0 | 37.8 | 2.8 | 10.9 |
|  |  | 4C | 2 | 1039.0 | 1062.3 | 1050.6 | 16.5 | 1.6 | -12.5 |
|  |  |  | 4 | 1135.0 | 1155.3 | 1145.2 | 14.4 | 1.3 | -4.6 |
|  |  |  | 24 | 1160.1 | 1211.2 | 1185.7 | 36.1 | 3.0 | -1.2 |
| 166 | 3410.0 | 22C | 2 | 3114.7 | 3400.2 | 3257.5 | 201.9 | 6.2 | -4.5 |
|  |  |  | 4 | 3208.6 | 3211.3 | 3209.9 | 1.9 | 0.1 | -5.9 |
|  |  |  | 24 | 3288.0 | 3445.5 | 3366.8 | 111.4 | 3.3 | -1.3 |
|  |  | 4C | 2 | 2813.7 | 3208.6 | 3011.2 | 279.2 | 9.3 | -11.7 |
|  |  |  | 4 | 3067.3 | 3263.0 | 3165.2 | 138.4 | 4.4 | -7.2 |
|  |  |  | 24 | 3139.8 | 3348.6 | 3244.2 | 147.6 | 4.6 | -4.9 |
| 177 | 826.9 | 22C | 2 | 767.3 | 856.6 | 811.9 | 63.1 | 7.8 | -1.8 |
|  |  |  | 4 | 863.5 | 856.6 | 860.0 | 4.9 | 0.6 | 4.0 |
|  |  |  | 24 | 537.1 | 547.4 | 542.2 | 7.2 | 1.3 | -34.4 |
|  |  | 4C | 2 | 694.2 | 664.1 | 679.2 | 21.3 | 3.1 | -17.9 |
|  |  |  | 4 | 726.3 | 766.3 | 746.3 | 28.2 | 3.8 | -9.7 |
|  |  |  | 24 | 702.3 | 681.2 | 691.7 | 14.9 | 2.2 | -16.3 |
| 187 | 3918.6 | 22C | 2 | 4482.6 | 4383.3 | 4433.0 | 70.2 | 1.6 | 13.1 |
|  |  |  | 4 | 4551.4 | 4638.2 | 4594.8 | 61.4 | 1.3 | 17.3 |
|  |  |  | 24 | 4512.2 | 4541.8 | 4527.0 | 20.9 | 0.5 | 15.5 |
|  |  | 4C | 2 | 4058.8 | 4738.0 | 4398.4 | 480.2 | 10.9 | 12.2 |
|  |  |  | 4 | 4050.1 | 4173.6 | 4111.9 | 87.4 | 2.1 | 4.9 |
|  |  |  | 24 | 4101.8 | 4217.4 | 4159.6 | 81.7 | 2.0 | 6.2 |

| Table S12 MMP-12 FRET Activity Assay  Intra-assay Imprecision of the Calibration Curve | | | | | | | |
| --- | --- | --- | --- | --- | --- | --- | --- |
| **Calibrator Concentration (pg/mL)** | **20,000** | **6,667** | **2,222** | **741** | **247** | **82** | **27** |
| Mean | 19875.0 | 6693.1 | 2140.7 | 826.0 | 235.8 | 68.1 | 22.3 |
| SD | 433.8 | 157.5 | 48.9 | 29.8 | 12.0 | 6.7 | 7.3 |
| % CV | 2.2 | 2.4 | 2.3 | 3.6 | 5.1 | 9.8 | 33.0 |
| % bias | -0.6 | 0.4 | -3.7 | 11.5 | -4.5 | -17.3 | -18.8 |
|  |  |  |  |  |  |  |  |
| Avg % CV | 3.6 | 5.5 | 8.1 | 7.1 | 7.2 | 16.0 | 37.8 |
| Avg % bias | -0.6 | 2.4 | -2.6 | 11.3 | 1.6 | -15.3 | -41.6 |

| Table S13 MMP-12 FRET Activity Assay  Inter-assay Imprecision of the Calibration Curve | | | | | | | |
| --- | --- | --- | --- | --- | --- | --- | --- |
| **Calibrator Concentration (pg/mL)** | **20,000** | **6,667** | **2,222** | **741** | **247** | **82** | **27** |
| Analytical Run # | Mean back-calculated concentrations from individual analytical runs | | | | | | |
| 1 | 19875.0 | 6693.1 | 2140.7 | 826.0 | 235.8 | 68.1 | 22.3 |
| 2 | 20012.5 | 6713.6 | 2123.1 | 850.0 | 255.3 | 63.6 | 4.3 |
| 3 | 19937.5 | 6679.9 | 2181.5 | 783.4 | 241.4 | 75.2 | 24.9 |
| 4 | 20009.1 | 6705.5 | 2119.9 | 853.3 | 253.5 | 61.1 | 9.5 |
| 5 | 19995.1 | 6699.1 | 2137.3 | 829.4 | 236.7 | 75.0 | 19.5 |
| Mean | 19965.8 | 6698.2 | 2140.5 | 828.4 | 244.5 | 68.6 | 16.1 |
| SD | 59.1 | 12.8 | 24.6 | 27.9 | 9.3 | 6.4 | 8.8 |
| % CV | 0.3 | 0.2 | 1.1 | 3.4 | 3.8 | 9.4 | 54.6 |
| % bias | -0.2 | 0.5 | -3.7 | 11.8 | -1.0 | -16.7 | -41.3 |

| **Table S14 MMP-12 FRET Activity Assay** | | | | |
| --- | --- | --- | --- | --- |
| **Dilution Linearity** | | | | |
| **Donor ID** | **diln** | **pg/mL** | **cv** | **% of previous diln** |
| 121 | 2 | 6933 | 14 |  |
|  | 4 | 8220 | 12 | 119 |
|  | 8 | 8147 | 2 | 99 |
|  | 16 | 7777 | 9 | 95 |
| 129 | 2 | 3528 | 4 |  |
|  | 4 | 4305 | 15 | 122 |
|  | 8 | 4273 | 6 | 99 |
|  | 16 | NA | NA | NA |
| 131 | 2 | 2840 | 6 |  |
|  | 4 | 3110 | 6 | 109 |
|  | 8 | 3901 | 16 | 125 |
|  | 16 | NA | NA | NA |
| 184 | 2 | 7366 | 25 |  |
|  | 4 | 7184 | 16 | 98 |
|  | 8 | 6170 | 8 | 86 |
|  | 16 | 4689 | 4 | 76 |

| **Table S15 MMP-12 FRET Activity Assay** | | | | | | | | |
| --- | --- | --- | --- | --- | --- | --- | --- | --- |
| **Donor Survey** | | | | | | | | |
| **Normal** | | | **Asthma** | | | **COPD** | | |
| **ids** | **avg** | **cv** | **ids** | **avg** | **cv** | **ids** | **avg** | **cv** |
| 150 | 213.1 | 6.7 | 151 | 2497.5 | 7.7 | 152 | 2226.3 | 6.4 |
| 157 | 1739.7 | 13 | 154 | 2361.8 | 7.1 | 153 | 2548.2 | 6.9 |
| 158 | 7626.1 | 15.6 | 156 | 1234.6 | 7.1 | 155 | 855.2 | 1.3 |
| 164 | 1538.5 | 0.5 | 163 | 1732.4 | 10 | 165 | 325.2 | 13.5 |
| 167 | 1809.3 | 15.5 | 166 | 1201 | 1.2 | 173 | 488.6 | 0.5 |
| 168 | 3016.1 | 13.2 | 169 | 2141.3 | 8.7 | 176 | 247.2 | 7.6 |
| 171 | 644.5 | 5.7 | 170 | 1160.3 | 8.9 | 180 | 1876.3 | 4.9 |
| 174 | 1552.4 | 3.3 | 178 | 2242.4 | 1.9 | 181 | 3076 | 10.8 |
| 175 | 597.1 | 6.8 | 182 | 875.7 | 6.6 | 183 | 1179.4 | 1 |
| 177 | 3227.9 | 5.6 | 184 | 9535.2 | 12.4 | 185 | 156.6 | 6 |
| 186 | 2261.3 | 9.2 |  |  |  |  |  |  |
| 187 | 1211.3 | 19.8 |  |  |  |  |  |  |
| 188 | 2364.6 | 13.1 |  |  |  |  |  |  |
| 189 | NA | NA |  |  |  |  |  |  |
| 190 | 1234.2 | 4.7 |  |  |  |  |  |  |
| 191 | 285 | 3.6 |  |  |  |  |  |  |
| 192 | 431.5 | 0.4 |  |  |  |  |  |  |
| 193 | 476.8 | 16 |  |  |  |  |  |  |
| Average | **1778.2** | **9** |  | **2498.2** | **7.2** |  | **1297.9** | **5.9** |

| Table S16 MMP-12 FRET Activity Assay  Intra / Inter-assay Imprecision of the QC Validation Samples | | | | | | | | | |
| --- | --- | --- | --- | --- | --- | --- | --- | --- | --- |
|  | **QC-low (Donor 192)** | | | **QC-mid ( Donor 158 )** | | | **QC-high ( Donor 151+spike )** | | |
| **Analytical Run #** | **PL080711** | **PL080721b** | **PL080722b** | **PL080811** | **PL080812** | **PL080813** | **PL080711** | **PL080721b** | **PL080722b** |
| Aliquot # |  |  |  |  |  |  |  |  |  |
| 1 | 645 | 579 | 696 | 5517 | 4732 | 4677 | 7219 | 10538 | 10223 |
| 2 | 570 | 431 | 870 | 4959 | 6349 | 5409 | 7128 | 9966 | 11468 |
| 3 | 621 | 601 | 440 | 4815 | 5001 | 3755 | 7399 | 9918 | 11235 |
| **Intra-assay analysis** |  |  |  |  |  |  |  |  |  |
| Mean | 612 | 537 | 669 | 5097 | 5361 | 4614 | 7249 | 10141 | 10975 |
| SD | 38 | 93 | 216 | 371 | 866 | 829 | 138 | 345 | 662 |
| % CV | 6 | 17 | 32 | 7 | 16 | 18 | 2 | 3 | 6 |
| **Avg % CV** | **19** | | | **14** | | | **4** | | |
| **Inter-assay analysis** | QC-low | | | QC-mid | | | QC-high | | |
| Mean | 606 | | | 5024 | | | 9455 | | |
| SD | 66 | | | 379 | | | 1956 | | |
| **% CV** | **11** | | | **8** | | | **21** | | |

| **Table S17 MMP-12 FRET Activity Assay** | | | | | | | | | | |
| --- | --- | --- | --- | --- | --- | --- | --- | --- | --- | --- |
| **Spike Recovery** | | | | | | | | | | |
| **Donor ID** | **Endogenous (pg/mL)** | **Spike** | **rep1** | **Rep2** | **Average** | **sd** | **% CV** | **Nominal (pg/mL)** | **% Recovery** | **% Bias** |
|  |  |  |  |  | **(pg/mL)** |  |  |  |  |  |
| 152 | 1965 | High | 17395 | 16468 | 16932 | 656 | 3.9 | 19685.7 | 78.9 | -21.1 |
|  |  | Low | 3089 | 3062 | 3075 | 19 | 0.6 | 1785.5 | 86.5 | -13.5 |
|  |  | Mid | 6283 | 6369 | 6326 | 61 | 1 | 5828.3 | 83.3 | -16.7 |
| 153 | 1810 | High | 19722 | 20041 | 19882 | 225 | 1.1 | 19685.7 | 93.3 | -6.7 |
|  |  | Low | 3431 | 3306 | 3368 | 88 | 2.6 | 1785.5 | 98.7 | -1.3 |
|  |  | Mid | 6781 | 6800 | 6790 | 13 | 0.2 | 5828.3 | 91.1 | -8.9 |
| 154 | 1042 | High | 21552 | 20695 | 21124 | 606 | 2.9 | 19685.7 | 102.4 | 2.4 |
|  |  | Low | 2903 | 2953 | 2928 | 35 | 1.2 | 1785.5 | 107.5 | 7.5 |
|  |  | Mid | 6857 | 6509 | 6683 | 246 | 3.7 | 5828.3 | 98.8 | -1.2 |
| 167 | 1122 | High | 22241 | 22118 | 22180 | 87 | 0.4 | 19685.7 | 107.2 | 7.2 |
|  |  | Low | 2742 | 2690 | 2716 | 37 | 1.4 | 1785.5 | 97.2 | -2.8 |
|  |  | Mid | 6827 | 6842 | 6834 | 11 | 0.2 | 5828.3 | 99.9 | -0.1 |

| **Table S18 MMP-12 FRET Activity Assay** | | | | | | | | | |
| --- | --- | --- | --- | --- | --- | --- | --- | --- | --- |
| **Short Term Stability** | | | | | | | | | |
| **Donor ID** | **Baseline (pg/mL)** | **temp (C )** | **Time (hours)** | **rep1** | **rep2** | **Average (pg/mL)** | **sd** | **%CV** | **% Change** |
| 151 | 1884 | 4 | 2 | 1801 | 1916 | 1858 | 81 | 4 | -1.3 |
|  |  |  | 4 | 2010 | 2007 | 2009 | 2 | 0 | 6.6 |
|  |  |  | 24 | 1297 | 1214 | 1256 | 59 | 5 | -33.3 |
|  |  | 22 | 2 | 1405 | 1935 | 1670 | 375 | 22 | -11.3 |
|  |  |  | 4 | 1415 | 2047 | 1731 | 447 | 26 | -8.1 |
|  |  |  | 24 | 1406 | 1194 | 1300 | 149 | 11 | -31 |
| 153 | 1800 | 4 | 2 | 2317 | 2574 | 2445 | 182 | 7 | 35.9 |
|  |  |  | 4 | 2142 | 2218 | 2180 | 54 | 2 | 21.1 |
|  |  |  | 24 | 1896 | 1597 | 1747 | 212 | 12 | -3 |
|  |  | 22 | 2 | 1518 | 2383 | 1951 | 612 | 31 | 8.4 |
|  |  |  | 4 | 1407 | 2176 | 1791 | 543 | 30 | -0.5 |
|  |  |  | 24 | 1645 | 786 | 1215 | 608 | 50 | -32.5 |
| 169 | 1846 | 4 | 2 | 1681 | 2205 | 1943 | 370 | 19 | 5.3 |
|  |  |  | 4 | 4203 | 4297 | 4250 | 67 | 2 | 130.3 |
|  |  |  | 24 | 4966 | 4704 | 4835 | 185 | 4 | 162 |
|  |  | 22 | 2 | 2243 | 3056 | 2649 | 575 | 22 | 43.5 |
|  |  |  | 4 | 4658 | 3717 | 4188 | 665 | 16 | 126.9 |
|  |  |  | 24 | 6278 | 5215 | 5746 | 751 | 13 | 211.4 |
| 178 | 3421 | 4 | 2 | 2275 | 3026 | 2650 | 532 | 20 | -22.5 |
|  |  |  | 4 | 2001 | 1907 | 1954 | 66 | 3 | -42.9 |
|  |  |  | 24 | 1648 | 1573 | 1610 | 53 | 3 | -52.9 |
|  |  | 22 | 2 | 2121 | 1673 | 1897 | 317 | 17 | -44.6 |
|  |  |  | 4 | 2483 | 1583 | 2033 | 636 | 31 | -40.6 |
|  |  |  | 24 | 1813 | 1693 | 1753 | 85 | 5 | -48.8 |

| **Table S19 MMP-12 FRET Activity Assay** | | | | | | | | |
| --- | --- | --- | --- | --- | --- | --- | --- | --- |
| **MMP-12 Freeze Thaw Stability** | | | | | | | | |
| **Donor ID** | **1F/T** | **F/T** | **rep1** | **rep2** | **Average (pg/mL)** | **sd** | **%CV** | **% Change** |
| 158 | 5096.7 | 2 | 3996.4 | 4422.3 | 4209.3 | 301.1 | 7.2 | -17.4 |
|  |  | 3 | 5557.5 | 4326.3 | 4941.9 | 870.6 | 17.6 | -3 |
|  |  | 4 | 4952.7 | 5429.1 | 5190.9 | 336.9 | 6.5 | 1.8 |
| 168 | 6065.7 | 2 | 4701.8 | 4632.5 | 4667.2 | 49 | 1 | -23.1 |
|  |  | 3 | 3952.5 | 4615.2 | 4283.9 | 468.6 | 10.9 | -29.4 |
|  |  | 4 | 4560 | 3851.4 | 4205.7 | 501 | 11.9 | -30.7 |
| 177 | 1289.8 | 2 | 1469.6 | 1720.3 | 1595 | 177.3 | 11.1 | 23.7 |
|  |  | 3 | 1280.9 | 1671 | 1475.9 | 275.8 | 18.7 | 14.4 |
|  |  | 4 | 1159.6 | 1274.6 | 1217.1 | 81.3 | 6.7 | -5.6 |
| 184 | 8194.8 | 2 | 6073.2 | 6124.2 | 6098.7 | 36 | 0.6 | -25.6 |
|  |  | 3 | 9160.2 | 8872 | 9016.1 | 203.8 | 2.3 | 10 |
|  |  | 4 | 9585.2 | 8323 | 8954.1 | 892.5 | 10 | 9.3 |
