# Supplementary figures and images for: Optimization of total protein and activity assays for the detection of MMP-12 in induced human sputum
Source: BMC Pulm Med. 2010 Aug 2;10:40. doi: 10.1186/1471-2466-10-40 (PMC2921351; doi:10.1186/1471-2466-10-40)

## Slide 1
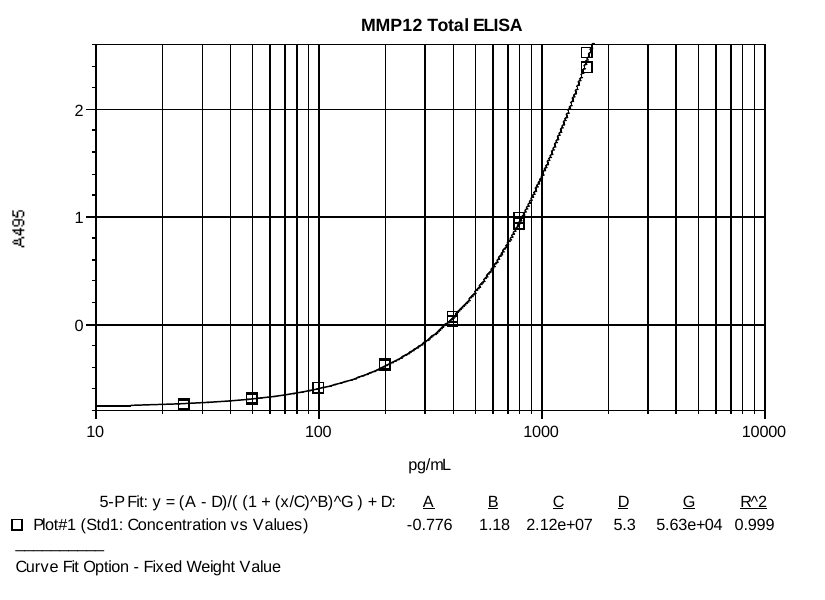

Supplement: Additional file 2 — Figure S1. An example standard curve for the MMP-12 total protein assay. [file 1471-2466-10-40-S2.PPT]

## Slide 1
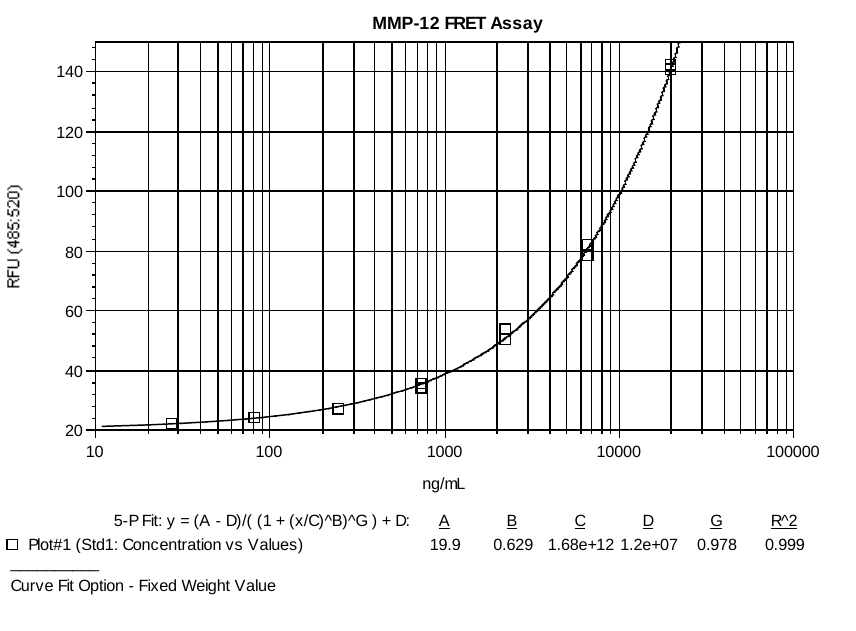

Supplement: Additional file 3 — Figure S2. An example standard curve for the MMP-12 FRET activity assay. [file 1471-2466-10-40-S3.PPT]
